# Non-Union Rate With Stand-Alone Lateral Lumbar Interbody Fusion

**DOI:** 10.1097/MD.0000000000000275

**Published:** 2014-12-02

**Authors:** Robert Watkins, Robert Watkins, Robert Hanna

**Affiliations:** From the Marina Spine Center, Marina del Rey, CA (RW, RW-III); and University of Southern California, Los Angeles, CA (RH).

## Abstract

Retrospective radiographic analysis.

To determine the fusion rate of stand-alone lateral lumbar interbody fusion (LLIF).

Biomechanical studies have indicated that LLIF may be more stable than anterior or transforaminal lumbar interbody fusion. Early clinical reports of stand-alone LLIF have shown success in obtaining fusion and indirectly decompressing nerve roots.

A consecutive case series of stand-alone LLIF was analyzed with chart and radiographic review. Non-union was determined by symptomatology consistent with non-union and absence of bridging bone on the CT scan.

Thirty-nine levels of stand alone LLIF were performed in 23 patients. Eleven patients received 1-level surgery, 7 patients received 2-level surgery, 3 patients received 3-level surgery, and 1 patient received 4-level surgery. Excluding 1 infected case, we analyzed 37 levels of stand alone LLIF in 22 patients. Non-union incidence was 7 levels in 6 patients. Non-union rate was 7/37 (19%) per level and 6/22 (27%) per patient.

While our study population was relatively low, a non-union rate of 19% to 27% is concerning for modern spine surgery. Currently in our practice, we occasionally still perform stand-alone LLIF utilizing 22 mm wide grafts in low-demand levels in non-smoking and non-osteoporotic patients. However, in a majority of patients, we provide supplemental fixation: bilateral pedicle screws in most patients and unilateral pedicle screws or spinous process plates in some patients.

## INTRODUCTION

In a lumbar fusion, performing a discectomy and interbody fusion can increase lordosis, indirectly decompress spinal nerves, increase stability and increase fusion rate. The direct anterior approach to the disc space (ALIF) probably allows for the best access and visualization of the disc space. The ALIF approach enables: resection of the anterior longitudinal ligament, wide discectomy, direct and indirect decompression of spinal nerves, insertion of large lordotic grafts, correction of deformity, and possible anterior instrumentation. The downsides of the ALIF approach include difficulty in obese patients, calcified vessels in the elderly, vascular injury, lymphatic injury, ureter injury, peritoneal and/or intestinal injury, ileus, wound hernia, sympathetic nerve injury, and retrograde ejaculation. From an implant perspective, resection of the anterior longitudinal ligament allows more lordotic grafts to be inserted, but results in partial loss of the ligamentous stability of the disc space. Furthermore, anterior instrumentation that is prominent outside the disc space may pose a risk to the surrounding vessels.

The lateral approach to lumbar spine is an alternative method to access the anterior column. Benefits of the lateral lumbar interbody fusion (LLIF) are large discectomy, bilateral annular release, preservation of the anterior longitudinal ligament, insertion of large grafts, correction of deformity, indirect decompression of spinal nerves, and possible lateral instrumentation. The downsides of the LLIF approach include superficial nerve injury, vascular injury, ureter injury, peritoneal and/or intestinal injury, ileus, psoas muscle injury, and lumbar plexus injury. From an implant perspective, preservation of the anterior longitudinal ligament restricts the amount of lordosis, but preserves some inherent ligamentous stability. A lateral plate outside the disc space may disturb the psoas muscle or lumbar plexus.

The transforaminal lumbar interbody fusion (TLIF) is performed with a posterior approach to the spine typically in conjunction with pedicle screw insertion and direct decompression of spinal nerves. However, the amount of discectomy, endplate preparation, annular release, graft size, and lordosis is typically is limited with TLIF compared to ALIF and LLIF.^1,2^

Recently, LLIF has significantly increased in popularity and usage worldwide. The relatively low morbidity of the lateral approach has allowed the surgery to be done with minimal hospital stay and even as an out-patient procedure. The fundamental issue is whether a LLIF stand-alone graft is sufficient to obtain fusion.

## MATERIALS AND METHODS

A consecutive case series of stand-alone LLIF was analyzed with chart and radiographic review, by Author B. The approach to the lateral disc space was done with a tubular retractor and psoas-splitting technique. Disc space preparation was done under anterior–posterior (AP) fluoroscopy with special attention to release the contralateral annulus and preservation of the bony endplates. To determine the appropriate height of the interbody grafts, trials were inserted under AP fluoroscopy. In general, the trial was deemed appropriate in height if it distracted a collapsed disc space and required a slap hammer for removal. The implant consisted of 18 mm wide (AP) PEEK grafts (RTI Surgical, Alachua, FL and Medtronic, Memphis, TN) with 4 mg of Infuse (Medtronic) and bone graft substitute (NanOss, RTI Surgical, Alachua, FL and MasterGraft, Medtronic). The use of PEEK grafts and Infuse from a lateral approach to the lumbar spine is off label according to the FDA. The choice of Infuse instead of autograft was based on reports of similar fusion rates without the morbidity of the autograft harvest site.^3^ All patients received an informed consent and agreed to the procedure. Patients were discharged home when they were ambulating independently, showing signs of bowel motility, and comfortable on oral pain medications. Typically, patients were discharged home 1 to 2 days post-operative.

Patients were routinely followed every 3 months with clinic evaluation and radiographs. Patient self-assessment including visual analog scale (VAS) at pre-operative and post-operative visits. Patients who were symptomatic underwent CT scan to check for bony union. Non-union was determined by symptomatology consistent with non-union and absence of bridging bone on the CT scan.

## RESULTS

**FIGURE 1:**
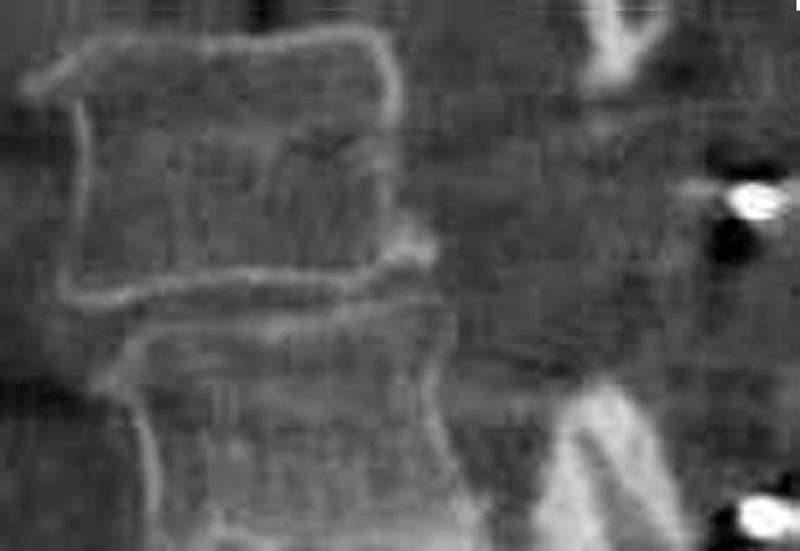
Pre-operative CT scan showing L2 to L3 degeneration and stenosis adjacent to previous L3 to S1 fusion.

**FIGURE 2:**
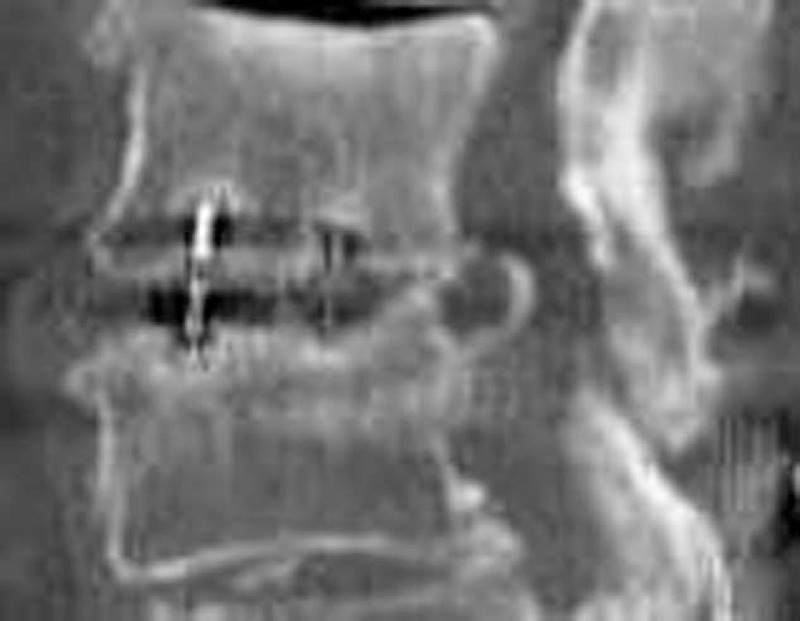
After 3 month pain-free interval, neurogenic claudication symptoms returned. Six-month postoperative CT scan shows subsidence of graft and ossification of annular bulge causing stenosis.

Between May 2008 and February 2012, 39 levels of stand-alone LLIF were performed in 23 patients. Eleven patients received 1-level surgery, 7 patients received 2-level surgery, 3 patients received 3-level surgery, and 1 patient received 4-level surgery. The results showed a total of 7 non-union levels in 6 patients. The breakdown of non-union levels per type of surgery was: 1-level non-union in 3 patients with 1-level surgery, 1-level non-union in 1 patient with 2-level surgery, 2-level non-union in 1 patient with 2-level surgery, 1-level non-union in 1 patient with 3-level surgery. An example of a non-union patient is Figures 1 and 2. One patient developed an early post-op infection in both levels of a 2-level surgery.

Excluding the infected patient, we analyzed 37 levels of stand alone LLIF in 22 patients. Non-union incidence was 7 levels in 6 patients. Non-union rate was 7/37 (19%) per level and 6/22 (27%) per patient.

Demographics comparing the fusion versus the non-union patients are in Table 1. Of note, there were three patients with non-unions that did not smoke or have osteoporosis. There was one patient who had two levels of non-union that smoked and had after period.

**TABLE 1:**
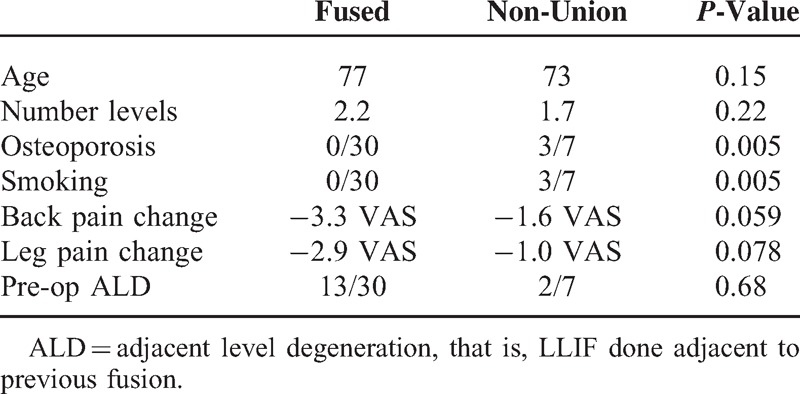
Demographics of Patient Population by levels

The results of the VAS scores are in Table 2. Of significance, the fused patients had significantly less back pain and leg pain post-operatively compared to the non-union patients.

**TABLE 2:**
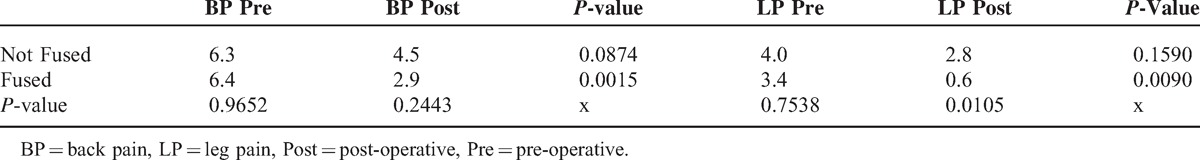
Pre and Post-Op Pain Scores by Fusion Status, by Patient

The change of back pain and leg pain from pre-operative to post-operative did not show a significant difference between the fused and non-union patients (Table 3). The small sample size probably accounts for the lack of statistical significance.

**TABLE 3:**
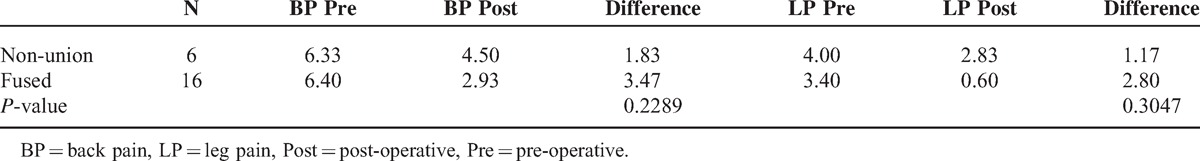
Difference in Pain Scores From Pre-operative to Post-operative

## DISCUSSION

The goal of most fusion surgeries is to decompress nerve roots and obtain an anatomic fusion with the least invasive surgery. Stand-alone interbody fusions through an anterior or lateral approach may be less invasive and have less muscle trauma than a traditional posterior approach. The lesser trauma of the anterior or lateral approach is only of benefit if the interbody fusion accomplishes the goals of the surgery. The interbody grafts must successfully decompress nerve roots and provide sufficient stability to obtain fusion.

The lateral approach has potential benefits over the anterior approach because there may be less peritoneal and bowel mobilization, as well as, less vascular mobilization for accessing L1 to L5. The incidence of wound hernias also may be less with a lateral approach versus an anterior approach. A lateral approach may not require the assistance of a vascular surgeon to the same degree as an anterior approach.

There are some clinical studies showing the ability of LLIF grafts to indirectly decompress nerve roots. Analysis of pre-operative and post-operative CT scans by Kepler et al^4^ showed that placement of a LLIF graft increased foraminal area by an average of 35%. MRI measurement after LLIF and percutaneous pedicle screws by Elowitz et al^5^ found that the area of the dural sac increased by an average of 143%. Oliveira et al^6^ demonstrated the ability of LLIF to increase disc height (42%), foraminal height (14%), foraminal area (25%), and central canal diameter (33%). Watkins et al^2^ demonstrated that LLIF with pedicle screws increased disc height by an average of 2.0 mm.

The LLIF grafts provide initial distraction and indirect decompression of nerve roots but if the surgical motion segment does not have sufficient stability then fusion may not occur. In general, biomechanical studies show that stand-alone lateral grafts significantly increase the stability over the pre-surgical intact segment. However, there is some conflicting data.

Kretzer et al^7^ reported biomechanical testing on 7 cadaveric specimens undergoing LLIF. They showed that stand-alone cage decreased ROM in all testing modes, and that the addition of facet screws and pedicle screws did not show a statistically significant improvement in stability.

Kim et al^8^ compared the stability ALIF versus LLIF femoral ring allografts in 16 cadavers. They did not find a statistically significant difference in the ROM of the intact disc versus the ALIF or LLIF grafts. However, most surgeons utilize a much wider medial–lateral LLIF graft than a femoral ring allograft, therefore, this study may not represent the majority of LLIF procedures.

Laws et al^9^ compared the biomechanical differences between ALIF and LLIF with and without supplemental instrumentation in 8 cadaveric specimens. They found that compared to the intact state, stand-alone LLIF significantly reduced ROM in flexion, extension, and lateral bending. Stand-alone ALIF did not stabilize motion segments relative to the intact state in any direction. No differences were observed between ALIF and LLIF groups supplemented by bilateral pedicle screws.

Cappuccino et al^10^ performed flexibility testing on 10 cadaveric specimens after LLIF constructs and compared the results to literature-reported biomechanical studies of other lumbar fusion constructs. They found that the LLIF construct provided the largest stand-alone reduction in ROM compared with literature-reported ALIF and TLIF constructs, especially in flexion–extension. Supplemental bilateral pedicle screw-based fixation provided the overall greatest reduction in ROM, similar among all interbody approach techniques. Lateral fixation and unilateral pedicle screw fixation provided intermediate reductions in ROM.

Biomechanical studies suggest that LLIF may be more stable than ALIF as a stand-alone construct. Clinical studies of stand-alone ALIF have reported non-union rates as high as 35% to 56%.^11,12^ Most stand-alone ALIF constructs used currently in the United Sates consist of supplemental fixation, either separate anterior plate or screws built into the cage. Clinical studies of stand-alone LLIF have shown various results.

Youssef et al^13^ reported 15 patients with stand-alone LLIF and 69 patients with supplemental fixation. At 6-month follow-up, they reported 68 demonstrated solid fusion on CT and dynamic radiographs in both groups combined. Two patients were lost to follow-up, and the remaining 14 had not completed remaining follow-up.

Sharma et al^14^ reported on 10 patients who underwent stand-alone LLIF at 16 levels. Two patients showed progressive subsidence, which may indicate a 20% non-union rate per patient (2/10). They reported 1 patient with 4-level non-union, which may indicate a 25% non-union rate per level.

Marchi et al^15^ reported on 52 patients with single-level grade I/II degenerative spondylolisthesis without significant instability undergoing stand-alone LLIF. They showed 86.5% solid fusion at 24-month evaluation. However, analysis of the reported data reveals that at 12-month evaluation only 67.3% showed evidence of fusion. A stand-alone graft that lacks supplemental fixation would not seem to gain stability after the 12-month mark to promote the fusion process. Furthermore, they reported that the 7 levels that were deemed to have incomplete bone growth at 24-months (13.5% non-unions) did not require revision surgery. Yet, 7 other cases did require revision surgery. Five revision cases had experienced subsidence with instability/restenosis. The 2 other cases required revision surgery because indirect decompression was not achieved. Including these revision cases with the reported non-union cases at 24-month evaluation, may lead to a failed stand-alone fusion rate of 26.9% (14/52).

The same group of surgeons, Pimenta et al,^16^ reported a prospective study of 30 patients undergoing stand-alone LLIF at L4–5 for DDD. They reported a 100% fusion rate at 36 months. However, their data shows insufficient indirect decompression in 7%, subsidence in 17%, and re-operation consisting of decompression and addition of pedicle screws in 13%. Considering the re-operation rate with addition of pedicle screws, the maximum conceivable fusion rate for the stand-alone LLIF was 87%, and this does not account some of the subsidence patients.

Recently, the same group of surgeons reported less subsidence using 22 mm versus 18 mm wide grafts in stand-alone LLIF.^17^ Overall, they reported a 91% fusion rate at 12-month follow-up. However, 10 (13.5%) of 74 patients required revision surgery consisting of decompression and pedicle screws on average of 10.4 weeks after surgery. In the discussion, they focus on the need for revision surgery due to inadequate indirect decompression. However, the lack of stability of the stand-alone LLIF may have equally contributed to the post-operative symptoms requiring revision surgery. The lack of stability may have been indicative of a developing non-union. It is not clear how they could have 13.5% revision surgery, but only 9% non-union.

This last study raises an important topic in LLIF surgery: the relationship between indirect decompression and stability. The LLIF graft alone has been shown to indirectly decompress nerve roots: most reliably in the foramen, but also in the central canal. The failure of a stand-alone LLIF graft to decompress a nerve root may be due to either inadequate distraction or failure to immobilize the motion segment. Post-operative subsidence of the LLIF graft may be due to violation of the bony endplates at the time of surgery or inadequate stabilization allowing micro-motion between the graft and endplates resulting in osteolysis. A LLIF graft may better be able to indirectly decompress nerves if it is supplemented with solid fixation (ie, pedicle screws) than if used as a stand-alone device. Furthermore, failure of a stand-alone graft to indirectly decompress nerves may not necessarily be due to subsidence as a result of an intra-operative violation of endplates, but may be due to excessive motion from a lack of adequate stability. Indirect decompression and stability are inter-related. Therefore, when interpreting results of studies, revision surgery and non-union rates need to be analyzed in relation to each other.

Non-union rates in all types of lumbar fusions have been reported in the 0% to 40% range.^18^ With modern techniques such as interbody fusions, pedicle screw fixation, and bone graft substitutes, non-union rates are typically in the 0% to 10% range.^19–21^ While our study population was relatively low, a non-union rate of 19% to 27% is concerning for modern spine surgery. Of note, all of the fused patients did not smoke or have osteoporosis. Currently in our practice, we occasionally still perform stand-alone LLIF utilizing 22 mm wide grafts in low-demand levels in non-smoking and non-osteoporotic patients. However, in a majority of patients, we provide supplemental fixation: bilateral pedicle screws in most patients and unilateral pedicle screws or spinous process plates in some patients.

## CONCLUSION

Our study of stand-alone LLIF shows a non-union rate of 19% per level and 27% per patient.
